# Role of the HIV-1 envelope transmembrane domain in intracellular sorting

**DOI:** 10.1186/s12860-018-0153-4

**Published:** 2018-03-15

**Authors:** Jackie Perrin, Aurélie Bary, Alexandre Vernay, Pierre Cosson

**Affiliations:** Department of Cell Physiology and Metabolism, Faculty of Medicine, University of Geneva, 1 rue Michel Servet, 1211 Geneva 4, Switzerland

**Keywords:** Secretory pathway, Transmembrane domain, Envelope protein, gp160, HIV-1, Endoplasmic reticulum

## Abstract

**Background:**

The envelope protein of lentiviruses are type I transmembrane proteins, and their transmembrane domain contains conserved potentially charged residues. This highly unusual feature would be expected to cause endoplasmic reticulum (ER) localization. The aim of this study was to determine by which means the HIV-1 Env protein is transported to the cell surface although its transmembrane domain contains a conserved arginine residue.

**Results:**

We expressed various chimeric proteins and analyzed the influence of their transmembrane domain on their intracellular localization. The transmembrane domain of the HIV-1 Env protein does not cause ER retention. This is not due to the presence of conserved glycine residues, or to the position of the arginine residue, but to the length of the transmembrane domain. A shortened version of the Env transmembrane domain causes arginine-dependent ER targeting. Remarkably, the transmembrane domain of the HIV-1 Env protein, although it does not confer ER retention, interacts efficiently with negatively charged residues in the membrane.

**Conclusion:**

These results suggest that the intrinsic properties of the HIV-1 Env transmembrane domain allow the protein to escape ER-retention mechanisms, while maintaining its ability to interact with cellular proteins and to influence cellular physiology.

## Background

Transmembrane proteins present at the surface of eukaryotic cells are initially inserted in the membrane of the endoplasmic reticulum (ER), from where they are transported to the Golgi apparatus and ultimately to the cell surface. Intracellular transport along the secretory pathway is coupled with sorting of proteins and lipids. As a consequence, each individual protein can eventually be found in the ER, in the Golgi apparatus, or at the cell surface. This ensures the proper localization of individual proteins in the compartment where their function is required (e.g. the ER for the signal peptidase, or the surface for the transferrin receptor). It also avoids the transport to the cell surface of proteins that are misfolded or incompletely assembled, and participates in the quality control of secreted proteins (reviewed in [1, 2]).

To ensure its proper sorting, each protein inserted in the ER exhibits specific motifs that can be recognized by the cellular transport and sorting machinery. These sorting motifs can be found in luminal domains (e.g. a KDEL ER-localization sequence), in cytosolic domains (e.g. a C-terminal KKXX ER-localization sequence) or in transmembrane domains (TMDs) (reviewed in [3]). The best-characterized ER localization motifs found in TMDs are potentially charged residues found in a number of type I transmembrane proteins. Charged residues are for example found in the TMDs of the various subunits of the T-cell receptor, and of a collection of receptors associated with DAP10, DAP12 or the FcRγ chain [4]. Typically a single charged residue in a TMD is sufficient to cause ER retention unless it is masked by the assembly and folding of protein complexes [5]. The molecular machinery that ensures the recognition and ER localization of sorting motifs in TMDs is still largely unknown. To date, the best-characterized mechanism proposes that the Rer1 protein acts as a receptor recognizing specific features of TMDs and ensuring their localization in the ER [6].

The envelope protein (Env) of HIV-1 (human immunodeficiency virus type 1) exhibits a conserved arginine residue in the TMD of its gp41subunit. The role of this potentially charged residue is poorly understood. It has notably been proposed that the arginine and/or several conserved glycines may drive interactions with other cellular proteins [7]. In particular, peptides mimicking a portion of the HIV-1 Env TMD were shown to interact with subunits of the T-cell receptor and to modulate T-cell activation [8, 9]. Similar experiments suggested an interaction of the Env TMD with TLR2 in macrophages [10]. In addition, mutations in the Env TMD may influence its intracellular transport [11] or alter its ability to induce membrane fusion [12, 13].

The assembly of HIV-1 virions requires the presence of the processed Env protein at the cell surface of infected cells [14]. Indeed, it has been amply demonstrated that the Env protein is transported to the surface of a variety of cells [15, 16], although the presence of a charged residue in its TMD would be expected to ensure its localization in the ER. The aim of the current study is to study this apparent paradox and to determine to what extent the TMD of the HIV-1 Env protein influences its intracellular transport.

## Results

### Potentially charged residues in the TMD of lentiviral envelope proteins

**Fig. 1:**
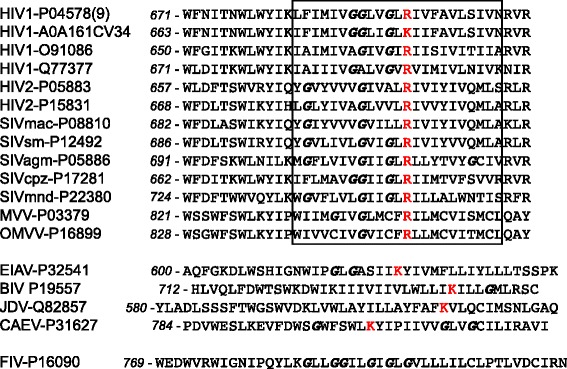
TMDs of lentiviral envelope proteins exhibit conserved charged residues. The sequence of the TMDs and surrounding region of several lentiviral envelope proteins are represented. Amino acid positions for each sequence are numbered in italic. The approximate position of the predicted TMDs is indicated. Potentially charged amino-acid residues are in red and glycine residues in italic. The Uniprot reference number of each protein is indicated

In almost every sequenced isolate of HIV-1, an arginine residue is positioned in the TMD of the Env protein (Fig. 1). In a few isolates (e.g. isolate 622,166-KT1247896.1) it is replaced, remarkably, with a lysine residue. This suggests that a positively charged residue at this position plays a critical role in the infectious cycle of HIV-1. An arginine or a lysine residue is also found in the TMD of the envelope protein of most other lentiviruses, notably HIV-2, simian (SIV) and bovine (BIV) immunodeficiency viruses, caprine arthritis/encephalitis virus (CAEV), Maedi visna ovine pneumonia virus (MVV) and equine infectious anemia virus (EIAV) (Fig. 1). In addition to a charged residue, many of these TMDs exhibit conserved glycine residues. In Feline Immunodeficiency virus (FIV), the TMD of the envelope protein contains no potentially charged residues, but exhibits six glycine residues (Fig. 1). The functional significance of these observations remains mostly unclear to date.

### The TMD of the HIV-1 Env protein does not confer ER retention

**Fig. 2:**
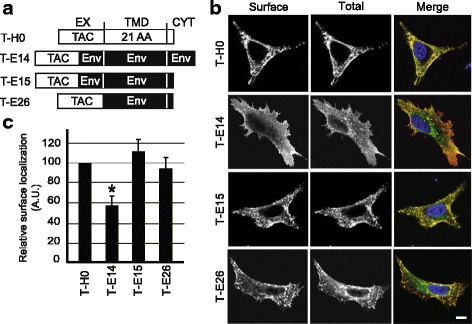
The HIV-1 Env TMD allows efficient cell surface localization. **a** The T-H0 chimera is composed of the extracellular domain of the Tac protein, a 21-hydrophobic residue transmembrane domain (TMD), and a short cytosolic domain (CYT). In T-E14, the Tac extracellular domain is fused to the Env extracellular juxtamembrane segment followed by the Env TMD and its complete cytoplasmic domain. The T-E15 protein exhibits a short truncated cytosolic domain. In T-E26, the Tac extracellular domain is fused directly to the Env TMD and a short cytoplasmic tail. Detailed amino acid sequences are described in Table 1. **b** Hela cell expressing the indicated Tac chimeric protein were labeled by immunofluorescence before (Surface) and after (Total) permeabilization, using antibodies specific for the Tac extracellular domain. All pictures were taken with the same settings with a confocal microscope (LSM700, Zeiss). Scale bar: 10 μm. **c** The amount of each Tac chimeric protein present at the cell surface was determined by dividing the surface fluorescence by the total fluorescence. For calibration, this ratio is set to 100 arbitrary units (A.U.) for T-H0. T-E14 is significantly less localized at the cell surface than T-H0 (*n* = 4; one-way analysis of variance: *p* < 0.01; *: post-hoc Tukey-Kramer *p* < 0.05)

The Env protein of HIV-1 is efficiently transported to the surface of infected cells despite the presence of a putatively charged arginine residue in its TMD which would be expected to cause ER retention [5]. In order to test if the Env TMD can affect its intracellular transport, we expressed fusion proteins composed of the extracellular domain of the Tac antigen fused to various TMDs (Fig. 2a and Table 1) and determined their intracellular localization. The Tac protein comprises an essentially hydrophobic 21 residues TMD (T-H0) and is readily transported to the cell surface, as revealed by successive labeling of surface and total Tac antigen in transfected cells (Fig. 2b). In the T-E14 protein, the Tac extracellular domain was fused to the Env TMD and cytoplasmic domain (Fig. 2a and Table 1). The Env extracellular juxtamembrane segment is very conserved in many lentiviral envelope proteins (Fig. 1) and it was also included in T-E14. The T-E14 protein was also abundantly present at the cell surface although a fraction of the protein was seen in intracellular compartments, most likely due to endocytic motifs in its cytosolic domain that have been shown to drive internalization from the cell surface to endosomes [17]. To quantify these observations, the surface level of Tac chimeric proteins was determined relative to the total expression level in individual cells. The T-E14 protein was indeed slightly less abundant at the cell surface than the T-H0 protein (Fig. 2c). In agreement with this interpretation the T-E15 protein, identical to T-E14 except for the deletion of its cytosolic domain, was almost exclusively localized at the cell surface (Fig. 2b and c). Similarly, the T-E26 protein where the Tac extracellular domain was fused to the TMD of Env without preserving the Env juxtamembrane region was mostly found at the cell surface (Fig. 2b and c). Together, these results indicate that despite the presence of a potentially charged arginine residue, the HIV-1 Env TMD does not target membrane proteins for efficient retention in the ER. This lack of ER retention is not due to an effect of the cytosolic or of the luminal juxtamembrane domains.

**Table 1:** Amino acids sequence of the transmembrane and cytosolic domains of the Tac chimeric proteins

Name	Luminal	Transmembrane	Cytoplasmic
T-H0	EY	QVAVAACVFLLIAVLLLSGLTWQ	RRQRKSRRTI
T-R8	EY	QVAVAACVRLLIAVLLLSGLTWQ	RRQRKSRRTI
T-H0KKxx	EY	QVAVAACVFLLIAVLLLSGLTWQ	RRQRLETFKKTN
T-E14	DL WLWYIK	IFIMIVGGLVGLRIVFAVLSIV	NRVR---LERILL
T-E15	DL WLWYIK	IFIMIVGGLVGLRIVFAVLSIV	NRVR
T-E26	DL	IFIMIVGGLVGLRIVFAVLSIV	NRVR
T-E28	DL	IMIVGGLVGLRIVFAVLSIV	NRVR
T-E30	DL	IVGGLVGLRIVFAVLSIV	NRVR
T-E34	DL	IVGGLVGLLIVFAVLSIV	NRVR
T-E39	DL	IFIMIVGGLVGLLIVFAVLSIV	NRVR
T-E41	DL	IFIMIVGGLVGLLIVFAVRSIV	NRVR
T-E42	DL	IFIMIVGGLVGLLIVFRVLSIV	NRVR
T-E43	DL	IFIMIVGGLVGLLIRFAVLSIV	NRVR
T-E44	DL	IFIMIVGGLVRLLIVFAVLSIV	NRVR
T-E45	DL	IFIMIVGGRVGLLIVFAVLSIV	NRVR
T-E46	DL	IFIMIVRGLVGLLIVFAVLSIV	NRVR
T-E59	DL	IFIMIVLLLVLLRIVFAVLSIV	NRVR
T-E60	DL	IFIMIVLLLVLLLIVFAVLSIV	NRVR

### The length of the HIV-1 Env TMD allows its exit from the ER

We next tried to determine why the arginine residue present in the Env TMD failed to confer ER localization. We tested specifically three possibilities: first, efficient transport to the cell surface may be due to an effect of conserved glycine residues in the Env TMD; second, the position of the charge in the Env TMD may be inadequate to cause ER retention; third the length of the Env TMD may cancel ER targeting by the arginine.

**Fig. 3:**
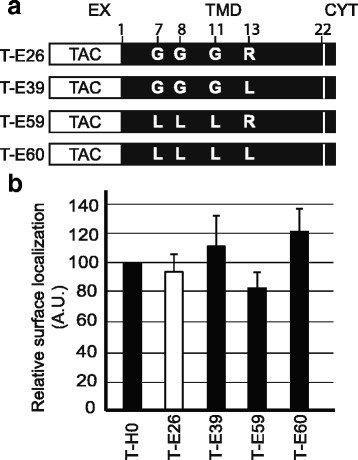
Glycine residues in the HIV-1 Env TMD do not affect its intracellular targeting. **a** Fusion proteins composed of the extracellular domain of the Tac protein, the transmembrane domain of Env protein (TMD) and a short cytosolic tail (CYT) are derived from the T-E26 chimera. In T-E39 the arginine present in the Env TMD is mutated to leucine. In T-E59 glycine residues in the Env TMD are mutated to leucines. Both mutations are combined in T-E60 (see also Table 1). **b** The fraction of each chimeric protein present at the cell surface was determined as described in the legend to Fig. 2. Statistical analysis revealed no significant differences from T-H0 (*n* = 3; one-way analysis of variance: *p* = 0.246)

The glycine residues present in the Env TMD are very conserved in many HIV-1 isolates, as well as in other lentiviruses (Fig. 1). They have been proposed to play a role in the trimerization of the HIV-1 Env TMD [7, 18], and this may in principle modify the recognition of the Env TMD by the cellular sorting machinery. To test this hypothesis, we compared the surface expression of T-E26, which comprises the Env TMD with that of T-E39 (where the arginine was mutated to leucine), of T-E59 (where the three glycine residues were mutated to leucines) and to T-E60 (where both the arginine and the glycine residues were mutated to leucines) (Fig. 3a). All of these chimeric proteins were present at similar levels at the cell surface (Fig. 3b), demonstrating that the arginine residue present in the Env TMD does not confer ER retention, even in the absence of glycine residues.

**Fig. 4:**
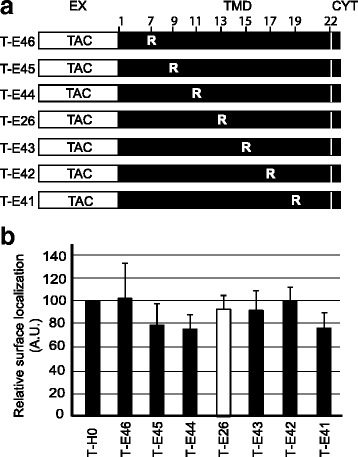
The position of the arginine residue in the HIV-1 Env TMD does not affect its intracellular targeting. **a** In the T-E26 chimera, an arginine is present in the Env TMD at position 14. A series of chimeric proteins where the arginine was moved to other positions were constructed (T-E41 to T-E46) (see also Table 1). Positions within the sequence are numbered from the luminal to the cytoplasmic end of the TMD. **b** The fraction of each chimeric protein present at the cell surface was determined as described in the legend to Fig. 2. Statistical analysis revealed no significant differences from T-H0 (n = 3; one-way analysis of variance: *p* = 0.77)

Since the position of potentially charged residues in a TMD can affect their ability to cause ER retention [5], we then studied the intracellular localization of fusion proteins where the arginine was placed at positions 7, 9, 11, 13, 15, 17 or 19 in the Env TMD (Fig. 4a and Table 1). This corresponded to a variety of positions at different levels in the lipid bilayer, as well as on various sides of the TM helix. For example positions 11 and 13 correspond to approximately the two opposite sides of the TM helix. All of the corresponding fusion proteins were efficiently expressed at the cell surface (Fig. 4b), indicating that the TMD of the Env protein does not cause ER retention independent of the position where an arginine residue is placed.

**Fig. 5:**
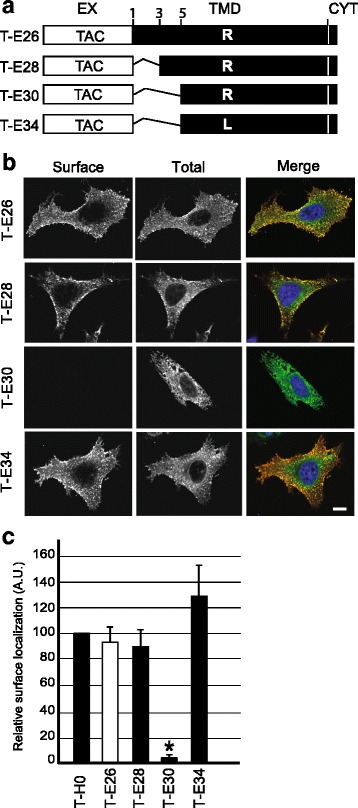
The intracellular targeting of the HIV-1 Env TMD is influenced by its length. **a** Compared to T-E26, two amino-acids were deleted from the Env TMD in T-E28, and four in T-E30. In addition, in T-E34 the arginine present in the TMD was mutated to a leucine residue (see also Table1). **b** Intracellular localization of each Tac chimeric protein was determined as described in the legend to Fig. 2. Scale bar: 10 μm. **c** The fraction of each chimeric protein present at the cell surface was determined as described in the legend to Fig. 2. T-E30 is significantly less localized at the cell surface than T-H0 (*n* = 4; one-way analysis of variance: *p* < 0.01; *: post-hoc Tukey-Kramer *p* < 0.05)

Previous studies have shown that the length of a TMD can also be an important element in its ability to confer specific intracellular location. For example, lengthening the TMD of a Golgi-targeted protein relocates it to the cell surface [19, 20], and similarly lengthening an ER-targeted TMD allows it to reach the cell surface [21]. There is however no algorithm capable of predicting with absolute precision the length of a TMD, and to define if a given TMD will be detected as unusually long by the intracellular sorting machinery. In order to test if the length of the Env TMD accounts for its ability to reach the cell surface, we expressed two chimeric proteins with a shortened TMD: the Env TMD of T-E26 was shortened by two residues in T-E28, and by four residues in T-E30 (Fig. 5a and Table 1). T-E28 was localized at the cell surface like T-E26 (Fig. 5b and c). T-E30 however was virtually depleted from the cell surface and localized almost exclusively in a reticular compartment akin to the ER (Fig. 5b and c). In order to verify if the intracellular retention of T-E30 was caused by the presence of a charged residue in its TMD, the arginine residue was replaced with a leucine in the fusion protein T-E34 (Fig. 5a and Table 1). T-E34 localized readily to the cell surface (Fig. 5b and c). These observations are also in agreement with a previous study showing that the unusually long TMD of the ε chain of the TCR does not cause retention in the ER despite the presence of a negatively charged residue [21].

**Fig. 6:**
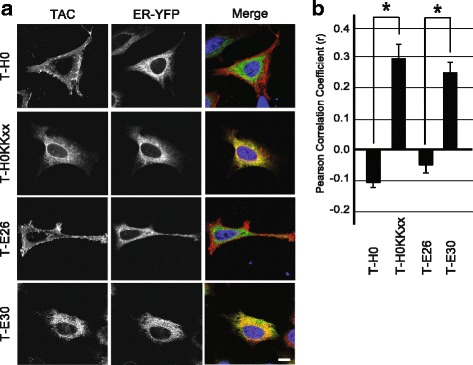
A shortened HIV-1 Env TMD acts as an ER-targeting motif. **a** Immunofluorescence microscopy of HeLa cells co-expressing various Tac fusion proteins (stained with an anti-Tac antibody) and a marker of the endoplasmic reticulum (ER-YFP). All pictures were taken with a confocal microscope (LSM700, Zeiss). Scale bar: 10 μm. **b** The colocalization of Tac proteins with the ER was quantified by measuring the Pearson’s correlation coefficient with Imaris software. T-E30 and T-H0KKxx are significantly localized in the ER (n = 4; *: Student’s *t-*test p < 0.01)

In order to ascertain that the intracellular T-E30 was localized in the ER, we coexpressed it with an ER-targeted fluorescent GFP protein. While T-H0 and T-E26 did not colocalize with the ER marker, T-H0KKxx which exhibits a cytosolic ER localization was mostly present in the ER, as well as T-E30 (Fig. 6a). This result was confirmed by calculating the Pearson correlation coefficient: the correlation coefficient between Tac fusion proteins and the ER marker was negative for T-H0 and T-E26, and positive for T-H0KKxx and T-E30 (Fig. 6b).

Together these results demonstrate that the length of the Env TMD is a key element allowing it to tolerate the presence of a charged residue while still escaping retention in the ER.

### The HIV-1 Env intramembrane arginine can interact with other proteins

**Fig. 7:**
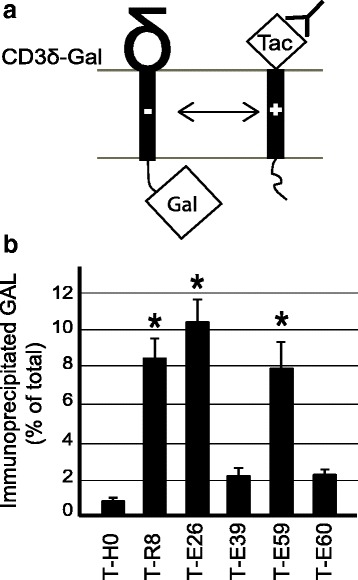
The HIV-1 Env TMD associates efficiently with a TMD containing a negative charge. **a** To reveal a putative interaction between TMDS, HeLa cells were co-transfected with plasmids encoding the δ chain of the CD3 receptor fused to the β-galactosidase (Gal) and various Tac fusion proteins. **b** Tac fusion proteins were immunoprecipitated and the amount of co-precipitated β-galactosidase activity assessed to reveal the degree of association with the δ chain of the CD3 receptor. The mean ± SEM of at least five independent experiments are indicated (one-way analysis of variance: p < 0.01; *: post-hoc Tukey-Kramer p < 0.05)

One mechanism by which the Env TMD could exit the ER despite the presence of an arginine would be if the length of the TMD somehow masked the arginine residue, making it invisible to the sorting machinery. To test this possibility, we coexpressed various Tac chimeric proteins and a protein (the δ subunit of the T-cell receptor) exhibiting a TMD containing an aspartic acid, and fused to β-galactosidase (Fig. 7a). We then immunoprecipitated the Tac antigen and determined the percentage of the β-galactosidase activity associated with it. As described previously [22], a TMD containing a charged residue (such as T-R8; Table 1) associated efficiently with a TMD containing a negative charge, whereas a hydrophobic TMD (T-H0) showed much less association (Fig. 7b). The Env TMD (T-E26) associated efficiently with its proposed partner, and this association was lost when the arginine was mutated to a leucine (T-E39 and T-E60), but did not decrease significantly when the glycine residues were mutated to leucines (T-E59)(Fig. 7b). These observations indicate that the arginine present in the Env TMD is readily available for interactions within the membrane.

## Discussion

This study was aimed at clarifying the potential role of the HIV-1 Env TMD in its intracellular transport. Our observations indicate that the charged residue present in the TMD of HIV-1 does not confer retention of the protein in the ER. According to our results, this is due to the fact that the TMD of HIV-1 is long enough to tolerate a charge and not be retained in the ER. Notwithstanding, the arginine residue is still capable of engaging into interactions within the membrane.

Our results shed new light on the molecular mechanisms that ensure recognition and ER retention of TMDs containing charges. Indeed, based on our current knowledge, two mechanisms can be envisaged to account for ER targeting by TMDs: TMDs containing a charged residue may be recognized by membrane receptors such as Rer1 that would ensure their proper intracellular sorting. Alternatively, structural and biophysical properties of a TMD, or interaction with various membrane lipids, may control its tendency to form aggregates, or its sorting to membrane subdomains enriched in specific lipids [3, 23]. According to this second model, a long TMD containing a charged residue may have biophysical properties akin to a shorter, hydrophobic TMD, while still exhibiting a charged residue capable of engaging into interactions in the membrane. In agreement with this model, our observations suggest that it is not the mere presence in the membrane of a charged residue capable of engaging into interactions that causes retention of a protein in the ER. Our observations rather suggest that the sorting machinery recognizes TMDs by a more complex mechanism, not based on simple interactions between charged residues within the membrane.

Importantly, throughout this text, we have made the choice of interpreting results based on the implicit assumption that localization of a protein in the ER reflects the presence of an ER retention or ER retrieval motif, while the absence of a retention motif would allow surface localization. An alternative interpretation would be that proteins are localized at the cell surface when they exhibit an ER-exit motif, and localized in the ER when they do not contain such a motif. Accordingly, a hydrophobic TMD, or a long TMD containing an arginine would represent an ER exit motif, while a short TMD would not direct for ER exit. For example, association with Erv14 may be favored with a long TMD but impossible with a short TMD containing an arginine [3, 24]. The two interpretations are not mutually exclusive: a long TMD may be transported efficiently at the cell surface due to both its association with Erv14 (facilitating ER exit) and its non-association with Rer1 (allowing escape from ER retrieval).

Our results bring two new elements to the discussion concerning the role of the charged residue in the HIV-1 Env TMD: first, despite the presence of an arginine residue the HIV-1 Env TMD does not per se cause retention in the ER; second, the arginine is available for interactions in the membrane. Our results are notably compatible with the notion that the Env TMD may interact with subunits of the T-cell receptor bearing TMDs with negatively charged residues, i.e. CD3 γ, δ, ε, and ζ. Indeed, in this study, the interaction partner that was proposed to the Env TMD was the CD3 δ subunit of the T-cell receptor, and our observations indicate that the HIV-1 Env TMD is capable of interacting specifically with this protein via its intramembrane arginine. Further experiments are needed to establish if, in the context of a full-length Env protein, the Env TMD is still available for efficient interactions with CD3 subunits. It remains also to be seen if in an HIV-infected T cell, the concentrations of the various proteins do allow such an interaction to effectively take place, and what its physiological consequences would be.

Interestingly, a recent study demonstrates that when an HIV-infected T cell is apposed to a non-infected T-cell, the Env protein at the surface of the infected cell interacts with the CD4 molecule on the uninfected cell [25]. Remarkably, this interaction results in the clustering of CD3 at the sites of contact, an observation compatible with the notion that the Env protein can directly interact with some elements of the T-cell receptor in an infected cell. Concomitant with this clustering, the T-cell receptor is activated, stimulating virus production, and facilitating virus spread to uninfected cells. We speculate that evolution may have favored an HIV-1 Env TMD that interacts with the T-cell receptor while still being able to escape ER retention and to reach the cell surface.

## Conclusions

The TMD of the HIV-1 Env protein contains a conserved charged residue. Contrary to several TMDs exhibiting the same feature, this TMD does not confer localization of the protein in the ER. Our results indicate that this is an intrinsic property of the HIV-1 Env TMD, which is long enough to tolerate a charge and not be retained in the ER. Despite the fact that it is not recognized by the ER targeting machinery, the Env TMD is still capable of interacting with other proteins within the membrane, and may by this means influence the physiology of infected cells.

## Methods

### Cell culture and media

HeLa cells were grown at 37 °C and 5% CO_2_ in Dulbecco’s modified Eagle’s medium (Gibco) containing 10% fetal bovine serum (Gibco) and 100 μg/ml of penicillin-streptomycin (Sigma). To express various fusion proteins, cells were transfected two days before the experiment using polyethylenimine (PEI) as previously described [26].

### Intracellular transport of tac chimeric proteins

We used a pCDM8-based vector containing the coding sequence of the α chain of the interleukin-2 receptor (Tac) as previously described [22]. In this study, all Tac fusion proteins were obtained by inserting the sequence coding for the TMD of interest (see Table 1) in the vector digested with *Bgl*II and *Xba*I.

Tac fusion proteins present at the cell surface were labeled with a mouse anti-Tac antibody (7G7) [27] for 15 min at 4 °C. Cells were then fixed for 10 min at room temperature in Phosphate Buffered Saline (PBS) containing 4% paraformaldehyde, washed with PBS containing 20 mM NH_4_Cl and incubated for 30 min at room temperature with an Alexa-Fluor-647-coupled anti-mouse-IgG antibody (Life Technologies, A21235) in PBS containing 0.2% Bovine Serum Albumine (PBS-BSA). Cells were then permeabilized for 10 min in PBS containing 0.2% saponin and labeled with 7G7 in PBS-BSA for 30 min. Finally, cells were incubated for 30 min with the Alexa-Fluor-488-coupled anti-mouse-IgG antibody (Life Technologies, A11029) before being mounted in Möwiol. Cells with a comparable total expression level were analyzed using a confocal microscope (LSM700, Zeiss).

When indicated, the ER was labeled by expressing a YFP-KDEL (ER-YFP, a kind gift of Nicolas Demaurex, University of Geneva, Switzerland).

Surface labeling was quantified with ImageJ software (http://rsb.info.nih.gov/ij/). The surface/total ratio was calculated for each individual cell using the surface fluorescence intensity and the total fluorescence intensity (in arbitrary units, using T-H0 as 100 a.u. for normalization). In each independent experiment, at least 15 cells were quantified.

### Association assays

Experiments were performed as previously described [28]. HeLa cells were co-transfected with Tac chimeric constructs and δ chain of the CD3 receptor fused to the β-galactosidase. Tac protein from the lysate was immunoprecipitated using protein A-agarose beads coated with 7G7 antibodies. The galactosidase activity is revealed upon addition of Chlorophenol Red-β-D-galactopyranoside and quantified by measuring absorbance at 600 nm. The percentage of β-galactosidase immunoprecipitated was determined.

## Abbreviations

ER: Endoplasmic reticulum.
HIV-1: Humain immunodeficiency virus type I.
TCR: T-cell receptor.
TMD: Transmembrane domain.
